# Smoking and Drinking Habits among the JPHC Study Participants at Baseline Survey

**DOI:** 10.2188/jea.11.6sup_44

**Published:** 2007-11-30

**Authors:** Tomotaka Sobue, Seiichiro Yamamoto, Shaw Watanabe

**Affiliations:** 1 Cancer Information and Epidemiology Division, National Cancer Center Research Institute.; 2 Department of Applied Bioscience, Tokyo University of Agriculture.

**Keywords:** smoking, drinking, type of alcohol

## Abstract

Smoking and drinking habits at baseline survey among 110,896 male and female residents aged 40-69 are reported as part of an ongoing prospective population-based cohort in 11 geographically diverse health centers in Japan. The age-adjusted proportion of current and former smokers was 54.8% and 21.8% in males and 8.3% and 2.1% in females, respectively. Mean age at initiation of smoking in males and females was 20.6 and 27.8 years of age, respectively. In males, the age-adjusted proportion of those who drink almost daily was 49.1% and that of those who drink almost never was 19.7%, while in females, it was 5.9% and 71.6%, respectively. When compared by health center, the proportion of male current smokers was lower in Miyako and Ishikawa, both located in Okinawa Prefecture, while in females the proportion was higher in urban areas, such as Katsushika and Suita. The proportion of those who drink almost daily in males was higher in Yokote, Kashiwazaki, Katsushika and Suita, and lower in Ishikawa and Miyako, but in Ishikawa and Miyako, the proportion of those who drink at social events was higher. In females, the proportion of those who drink almost daily was higher in urban areas. There was substantial variation in the types of alcohol beverages consumed by males. In contrast, alcohol consumption in females comprised mainly beer.

## INTRODUCTION

A number of epidemiological studies have suggested that smoking and drinking play important roles in the etiology of several cancers and cardiovascular diseases. Therefore, the distribution of smoking and drinking habits in a general cohort population is important in determining the generalizability of a given cohort.

The proportion of current smokers in Japan was monitored by Japan Tobacco and Salt Public Corporation from 1958 to 1984 and by Japan Tobacco Inc. from 1984^1^^)^. Also, since 1987, a survey of smoking habits was added to the National Nutrition Survey^2^^)^. In both surveys, the sex and age-specific proportion of current smokers has been reported annually. Drinking habits have also been monitored in the National Nutrition Survey^2^^)^.

There are several reports which have investigated the smoking and drinking habits in a specific population. However, these reports appear sporadically in the literature and are often difficult to compare because the investigation methods are not similar. Here, we report on baseline estimates of smoking and drinking habits in 11 public health centers. The data was obtained as part of the ongoing Japan Public Health Center Prospective Study on Cancer and Cardiovascular diseases (JPHC Study).

## MATERIALS AND METHODS

### Study population:

The study population, the study design, and other related details have been reported elsewhere in this journal. Table 1 shows the sex and age distribution of the study population. The study population in this analysis was 110,896 male and female respondents in Cohort I and II combined. In two districts, Katsushika and Suita1, only adults aged from 40 to 50 years were asked to participate, limiting the generalizability for these two districts.

**Table 1.** Sex and age distribution of study subjects and proportion of unknown cases on smoking and drinking habits.

		Cohort I (1990)						Cohort II (1993)								Total
	
Health Center		Ninohe	Yokote	Saku	Ishikawa	Katsushika*	Total	Kasama	Kashiwazaki	Tosayamada	Arikawa	Miyako	Suita1*	Suita2	Total	
Prefecture		Iwate	Akita	Nagano	Okinawa	Tokyo		Ibaraki	Niigata	Kochi	Nagasaki	Okinawa	Osaka	Osaka		
Male																
	40-49	2,133	2,605	2,604	2,655	1,318	11,315	4,008	415	1,404	1,552	1,554	1,226	542	10,701	22,016
	50-59	2,095	2,866	2,806	2,501	1,446	11,714	2,957	522	1,088	1,562	1,586	1,681	702	10,098	21,812
	60-69	–	–	–	–	–	–	2,488	583	1,069	1,869	1,639	–	850	8,498	8,498

	Total	4,228	5,471	5,410	5,156	2,764	23,029	9,453	1,520	3,561	4,983	4,779	2,907	2,094	29,297	52,326

%unknown	Smoking	0.2%	0.1%	0.0%	0.1%	0.0%	0.1%	0.6%	0.4%	0.2%	0.5%	0.4%	0.7%	0.0%	0.5%	0.3%
	Drinking	0.2%	0.2%	0.1%	0.2%	0.2%	0.2%	1.7%	3.1%	1.7%	3.5%	7.2%	0.9%	0.3%	2.6%	1.3%

Female																
	40-49	2,401	2,863	2,587	2,727	1,701	12,279	3,829	459	1,502	1,702	1,645	1,608	709	11,454	23,733
	50-59	2,472	3,420	2,890	2,570	2,283	13,635	2,896	552	1,219	2,144	1,711	1,974	836	11,332	24,967
	60-69	–	–	–	–	–	–	2,834	634	1,275	2,370	1,913	–	844	9,870	9,870

	Total	4,873	6,283	5,477	5,297	3,984	25,914	9,559	1,645	3,996	6,216	5,269	3,582	2,389	32,656	58,570

%unknown	Smoking	0.1%	0.1%	0.1%	0.1%	0.1%	0.1%	0.5%	4.7%	0.0%	0.5%	0.3%	0.1%	0.1%	0.5%	0.3%
	Drinking	0.3%	0.5%	0.1%	0.1%	0.2%	0.3%	1.1%	1.8%	1.0%	1.0%	1.9%	0.9%	0.2%	1.1%	0.7%

Total		9,101	11,754	10,887	10,453	6,748	48,943	19,012	3,165	7,557	11,199	10,048	6,489	4,483	61,953	110,896

* Study population consisted of only those aged 40 or 50 exactly in Katsushika and Suita1

### Smoking:

For both Cohort I and II, the questions on smoking consisted of current and former smoking habits, age at initiation of smoking, average number of cigarettes smoked per day and age at cessation of smoking for former smokers. Although the items were the same for both questionnaires, the structure and order of the questions were slightly different between Cohort I and II. In Cohort I, question A: “Have you ever smoked cigarettes,” was asked first and then question B: “Are you currently smoking cigarettes,” was asked for those who responded “Yes” to question A. Therefore, “nonsmoker”, “former smoker” and “current smoker” in this analysis was defined to be those who responded “No” to question A, those who responded “Yes” to question A and “No” to question B and those who responded “Yes” to question A and “Yes” to question B, respectively. All other combinations of responses including missing responses were defined as “unknown”.

In Cohort II, question B was asked first and then age at initiation of smoking and average number of cigarette smoked per day were asked for those who responded “Yes” to question B. For those who responded “No” to question B, age at initiation of smoking, average number of cigarettes smoked per day and age at cessation of smoking were asked if they had stopped smoking, without asking question A. Therefore, “nonsmoker” was defined as those who responded “No” to question B and no reply at all for the remaining items. “Former smoker” was defined as those who responded “No” to question B and responded to at least one of the following items: age at initiation of smoking, average number of cigarettes smoked per day, and age at cessation of smoking. “Current smoker” was defined those who responded “Yes” to question B. All other combinations of responses including missing responses were defined as “unknown”. The proportion of unknown cases for smoking habits is shown in Table 1. In all public health centers, the proportion was less than one percent except for females in Kashiwazaki. The subjects with an “unknown” smoking history were excluded from further tabulation.

### Drinking:

The questionnaires used in Cohort I and II differed to a larger extent with respect to the questions on alcohol. Basically, in both Cohort I and II, the frequency of alcohol intake was asked first, followed by the average amount of daily alcohol intake by type of alcohol. In Cohort I, current frequency of any alcohol intake was asked first using 6 categories; “almost never,” “1-3 times per month,” “1-2 times per week,” “3-4 times per week,” “5-6 times per week” and “daily.” For those who consumed alcohol at least once a week, the average amount of intake per day of 5 types of alcohol beverages (Japanese Sake, Shochu/Awamori (spirits), beer, whiskey, and others) was queried. In Cohort II, current drinking status was asked using 3 categories: “No”, “Stopped already” and “Yes.” For those who responded “Stopped already” or “Yes,” the frequency of regular alcohol intake was asked using 4 categories: “1-3 times per month,” “1-2 times per week,” “3-4 times per week,” “almost daily”. In addition, the average amount of daily intake was asked of all current and former drinkers for 8 types of alcohol beverages (Japanese Sake, Shochu (spirits), Awamori (spirits), beer (large bottle), beer (medium bottle), beer (small bottle), wine, and whiskey/brandy/vodka). In addition, the frequency of drinking at social events was asked as the average number of days per month, and average amount of intake per day was asked for 8 types of alcohol beverages in the same way as usual drinking.

Those who did not respond completely on these alcohol-related questions were defined as “unknown” for drinking habits. The proportion of unknown cases for drinking habits is also shown in Table 1. The proportions tended to be higher in the areas of Cohort II than in Cohort I, especially for males in Miyako, Arikawa and Kashiwazaki. The unknown cases were excluded from further tabulation.

In order to compare the frequency of alcohol intake among the 11 public health centers simultaneously, the category “5-6 times per week” and “daily” were combined as “almost daily” in Cohort I and those who responded “No” or “Stopped already” were combined as “almost never” in Cohort II. For these comparisons, data from drinking at social events were ignored in Cohort II, because subjects were not asked these questions in Cohort I. In order to compare the frequency of intake by type of alcohol beverages, the percent by type of alcohol beverage was calculated only for those who drink any type of alcohol at least 1-2 times per week or more. The data from drinking at social events was not used in these calculations.

Average ethanol intake per week was estimated by multiplying the frequency of intake, by the amount of ethanol for a specific type of alcohol beverage. Answers of “1-3 times per month,” “1-2 times per week,” “3-4 times per week,” “almost daily” were set to be 0.5, 1.5, 3.5, 6.0 times per week, respectively. Amount of ethanol in “Japanese Sake,” “Shochu/Awamori” (spirits), “beer,” “wine” and “whiskey/brandy/vodka” was set to be 23g/180ml, 36g/180ml, 23g/760ml, 3g/1glass and 10g/1glass, respectively. Average amount of ethanol intake was calculated for those who drink at least 1-2 times per week or more. The analysis was conducted for Cohort I and Cohort II separately, because data from drinking at social events was available only for Cohort II.

### Statistical Analysis:

All calculations were based on sex and 10-year age group. For the comparison of 11 Public Health Centers, age adjusted proportions were calculated by two age groups (40-49 and 50-59) using the 1985 Japanese population as the standard population. Ratios of these proportions were computed using the proportion for the 11 heath centers combined as the denominator.

## RESULTS

### Smoking:

Table 2 shows the proportions of current and former smokers by sex, age and public health center. The age-adjusted proportion of current and former smokers was 54.8% and 21.8% in males and 8.3% and 2.1% in females, respectively, for the 11 public health centers combined. In both males and females, the proportion of current smokers was lower in older age group. The proportion of former smokers was higher in the older age group in males, but there was no remarkable difference in females.

**Table 2.** Proportion of current and former smoker by sex, age and health center.

			Cohort I (1990)						Cohort II (1993)								Total
	
	Health Center		Ninohe	Yokote	Saku	Ishikawa	Katsushika*	Total	Kasama	Kashiwazaki	Tosayamada	Arikawa	Miyako	Suita1*	Suita2	Total	
	Prefecture		Iwate	Akita	Nagano	Okinawa	Tokyo		Ibaraki	Niigata	Kochi	Nagasaki	Okinawa	Osaka	Osaka		
Male																	
	Proportion of current smoker																
		40-49	59.6	60.8	60.2	46.6	61.0	57.1	63.3	61.5	62.8	60.9	43.8	60.6	56.5	59.3	58.2
		50-59	52.5	49.4	52.0	43.7	52.2	49.7	58.5	49.4	51.0	51.0	31.8	58.9	48.3	51.2	50.4
		60-69	–	–	–	–	–	–	49.2	47.4	43.3	46.5	32.3	–	43.2	43.9	43.9

		Age adjusted**	56.5	55.8	56.6	45.3	57.1	53.8	61.2	56.2	57.6	56.5	38.5	59.9	52.9	55.7	54.8
		Ratio***	1.03	1.02	1.03	0.83	1.04	0.98	1.12	1.03	1.05	1.03	0.70	1.09	0.97	1.02	1.00

	Proportion of former smoker																
		40-49	15.5	20.5	21.8	22.1	23.9	20.6	19.5	20.1	17.8	16.4	16.6	21.0	21.6	19.7	19.7
		50-59	19.4	24.8	27.4	30.3	28.4	26.1	22.7	25.4	21.0	23.3	17.1	23.9	28.9	22.5	24.4
		60-69	–	–	–	–	–	–	34.9	33.6	34.0	34.0	33.0	–	40.0	34.7	34.7

		Age adjusted**	17.2	22.4	24.3	25.7	25.9	23.0	20.9	22.4	19.2	19.4	16.8	22.3	24.8	20.9	21.8
		Ratio***	0.79	1.03	1.11	1.18	1.19	1.06	0.96	1.03	0.88	0.89	0.77	1.02	1.14	0.96	1.00

Female																	
	Proportion of current smoker																
		40-49	5.5	5.8	9.4	7.0	23.3	9.2	11.4	5.8	9.7	4.7	4.1	16.7	13.8	9.8	9.5
		50-59	3.3	2.7	5.0	8.5	16.1	6.6	8.4	1.5	5.1	3.6	4.1	12.4	10.3	7.0	6.8
		60-69	–	–	–	–	–	–	5.3	0.9	4.7	3.3	4.2	–	9.5	4.6	4.6

		Age adjusted**	4.5	4.4	7.5	7.7	20.1	8.1	10.1	3.9	7.7	4.2	4.1	14.8	12.3	8.6	8.3
		Ratio***	0.55	0.53	0.90	0.92	2.42	0.97	1.21	0.47	0.92	0.51	0.49	1.78	1.47	1.03	1.00

	Proportion of former smoker																
		40-49	1.6	1.5	2.6	2.1	9.2	3.0	1.3	1.1	1.3	0.8	0.6	4.6	3.0	1.7	2.4
		50-59	1.2	1.1	1.7	3.2	4.4	2.2	1.3	0.4	1.1	0.5	1.1	2.2	2.8	1.3	1.8
		60-69	–	–	–	–	–	–	1.4	0.2	1.6	1.0	1.6	–	5.0	1.6	1.6

		Age adjusted**	1.4	1.3	2.2	2.6	7.1	2.6	1.3	0.8	1.2	0.7	0.8	3.5	2.9	1.5	2.1
		Ratio***	0.67	0.62	1.03	1.21	3.32	1.24	0.61	0.37	0.57	0.31	0.38	1.66	1.36	0.71	1.00

* Study population consisted of only those aged 40 or 50 exactly in Katsushika and Suita1

** Age adjusted proportion by Japanese standard population (1985) using the data for age group 40-49 and 50-59

*** Ratio of the age-adjusted proportions setting that for 11 heath centers combined to be the denominator

In males, the proportion of current smokers was lower in Miyako and Ishikawa; both located in Okinawa Prefecture. The highest proportion of current smokers was observed in Kasama, but the differences among the health centers were not remarkable except for Miyako and Ishikawa. The proportion of former smokers was low in Ninohe, but the proportion of current smokers was high for this public health center. The proportion of former smokers tended to be slightly lower in areas of Cohort II than Cohort I, which may reflect a systematic bias as a result of using a different questionnaire.

In females, there was more variability in the proportion of current smokers by public health center. The proportion of current smokers was higher in urban areas, such as Katsushika and Suita. In the rural areas of Kashiwazaki, Miyako, Arikawa, Yokote and Ninohe, the proportion was low. In Saku, Ishikawa, Kasama and Tosayamada, which are a mixture of rural and suburban areas, the proportion was relatively high. As with males, the proportion of former smokers tended to be slightly lower in areas of Cohort II than Cohort I.

Table 3 shows the mean age at initiation of smoking and average number of cigarettes smoked per day among current smokers. Approximately, 1.0% of male and 3.5% of female smokers did not provide proper information on the above items and they were excluded from the analysis.

**Table 3.** Mean age at initiation of smoking and average number of cigarettes smoked perday by sex, age and health center.

			Cohort I (1990)						Cohort II (1993)								Total
	
	Health Center		Ninohe	Yokote	Saku	Ishikawa	Katsushika*	Total	Kasama	Kashiwazaki	Tosayamada	Arikawa	Miyako	Suita1*	Suita2	Total	
	Prefecture		Iwate	Akita	Nagano	Okinawa	Tokyo		Ibaraki	Niigata	Kochi	Nagasaki	Okinawa	Osaka	Osaka		
Male																	
	Mean age at initiation of smoking																
		40-49	20.8	20.4	20.0	20.9	19.5	20.4	19.7	20.4	19.6	20.0	21.2	19.4	19.9	20.0	20.2
		50-59	21.6	21.4	21.1	21.3	20.4	21.4	20.3	21.5	20.6	21.0	23.6	19.8	20.8	20.9	21.1
		60-69	–	–	–	–	–	–	21.1	21.7	20.7	21.1	23.3	–	20.8	21.6	21.4

		Age adjusted**	21.2	20.8	20.5	21.1	19.9	20.8	20.0	20.9	20.1	20.4	22.3	19.6	20.3	20.4	20.6
		Ratio***	1.03	1.01	1.00	1.02	0.97	1.01	0.97	1.02	0.98	0.99	1.08	0.95	0.99	0.99	1.00

	Average number of cigarettes smoked per day																
		40-49	22.7	22.9	24.2	22.7	24.7	23.4	25.1	24.4	26.6	26.6	25.4	26.9	27.9	25.9	24.6
		50-59	21.0	20.7	21.5	21.8	24.3	21.6	23.5	22.1	23.3	24.0	25.0	27.8	26.2	24.6	23.0
		60-69	–	–	–	–	–	–	18.6	17.8	19.3	19.1	18.8	–	19.5	18.9	18.9

		Age adjusted**	22.0	21.9	23.0	22.3	24.5	22.6	24.4	23.4	25.1	25.5	25.2	27.3	27.2	25.3	23.9
		Ratio***	0.92	0.92	0.96	0.93	1.03	0.95	1.02	0.98	1.05	1.07	1.06	1.14	1.14	1.06	1.00

Female																	
	Mean age at initiation of smoking																
		40-49	26.4	28.4	26.3	27.3	21.9	28.1	25.5	24.1	24.3	23.3	25.3	21.9	24.3	26.8	25.3
		50-59	33.9	31.5	32.2	32.5	28.2	25.6	32.3	29.6	28.1	30.0	31.7	27.6	29.4	24.5	31.0
		60-69	–	–	–	–	–	–	36.0	37.4	31.4	30.9	34.8	–	32.5	30.2	33.6

		Age adjusted**	29.7	29.8	28.9	29.6	24.7	27.0	28.5	26.5	26.0	26.3	28.1	24.4	26.6	25.8	27.8
		Ratio***	1.07	1.07	1.04	1.06	0.89	0.97	1.02	0.95	0.93	0.94	1.01	0.88	0.95	0.93	1.00

	Average number of cigarettes smoked per day																
		40-49	12.7	13.2	12.1	15.3	14.2	13.6	16.0	12.0	16.0	17.9	16.4	14.6	14.7	15.6	14.6
		50-59	11.2	13.3	13.0	14.9	16.1	14.6	15.2	15.8	14.0	13.6	15.6	16.5	15.6	15.4	15.0
		60-69	–	–	–	–	–	–	13.4	12.0	13.8	12.3	12.6	–	13.7	13.2	13.2

		Age adjusted**	12.0	13.2	12.5	15.1	15.0	14.0	15.6	13.7	15.1	16.0	16.0	15.4	15.1	15.5	14.8
		Ratio***	0.81	0.90	0.85	1.02	1.02	0.95	1.06	0.93	1.02	1.08	1.09	1.04	1.02	1.05	1.00

* Study population consisted of only those aged 40 or 50 exactly in Katsushika and Suita1

** Age adjusted proportion by Japanese standard population (1985) using the data for age group 40-49 and 50-59

*** Ratio of the age-adjusted proportions setting that for 11 heath centers combined to be the denominator

The average age at initiation of smoking in males and females was 20.6 and 27.8 years of age, respectively. In males, there was little variation on the mean age at initiation of smoking by cohort age group, while in females the average age at initiation of smoking was higher for the older age group. There was no remarkable difference in the average age at initiation of smoking in males across public health centers, although it was slightly higher in Miyako. In females, however, the mean age at starting to smoke was lower in the two urban areas of Katsushika and Suita.

The average number of cigarettes smoked per day tended to decrease in the older age group in males, while there was little difference in females. Across public health centers, the average number smoked in males was higher in Suita and lower in Ninohe and Yokote. In females, the average number was higher in Arikawa and Miyako and lower in Nihohe and Saku. In both males and females, the average number of cigarette smoked per day tended to be slightly higher in areas of Cohort II public health centers.

### Drinking:

Table 4a shows the distribution of alcohol consumption in males by age and health center. Overall, the age-adjusted proportion of those who drink almost daily was 49.1% and of those who drink almost none was 19.7% for males. In the older age group, the proportion of those who almost never drink was higher than of those who drink occasionally, while that of those who drink almost daily did not change remarkably across age groups.

**Table 4a.** Distribution for frequency of alcohol intake by age and health center, male.

		Cohort I (1990)						Cohort II (1993)								Total
	
Health Center		Ninohe	Yokote	Saku	Ishikawa	Katsushika*	Total	Kasama	Kashiwazaki	Tosayamada	Arikawa	Miyako	Suita1*	Suita2	Total	
Prefecture		Iwate	Akita	Nagano	Okinawa	Tokyo		Ibaraki	Niigata	Kochi	Nagasaki	Okinawa	Osaka	Osaka		
Age	Frequency															
40-49	Almost None	21.3	12.5	18.1	23.6	18.4	18.7	20.5	14.1	16.0	22.7	9.8	15.0	15.0	17.5	18.2
	1-3 / month	9.4	6.3	9.5	19.6	6.9	10.8	7.8	7.2	9.0	12.3	15.8	6.2	3.7	9.3	10.1
	1-2 / week	8.6	6.7	8.1	18.4	8.0	10.3	9.0	6.2	10.0	9.0	23.0	10.1	8.9	11.1	10.7
	3-4 / week	11.5	9.3	9.8	15.7	9.7	11.4	13.5	9.9	14.0	13.9	23.9	10.8	11.1	14.5	12.9
	almost daily	49.2	65.2	54.5	22.7	57.0	48.8	49.3	62.6	51.0	42.0	27.5	57.9	61.2	47.6	48.2

50-59	Almost None	25.7	15.6	20.1	30.7	17.0	21.9	24.8	13.3	22.2	30.9	15.7	15.3	19.3	21.5	21.7
	1-3 / month	9.7	5.6	8.2	17.7	6.5	9.6	6.7	4.6	9.9	7.9	13.4	3.7	3.3	7.4	8.6
	1-2 / week	8.7	4.8	6.4	14.8	6.8	8.3	7.2	5.8	10.1	7.7	16.1	6.8	7.1	8.8	8.5
	3-4 / week	9.4	7.5	7.3	14.3	9.5	9.5	11.3	8.9	11.2	10.5	19.5	11.0	15.0	12.5	10.9
	almost daily	46.4	66.5	58.0	22.4	60.2	50.7	49.9	67.5	46.6	43.0	35.3	63.2	55.3	49.9	50.3

60-69	Almost None	–	–	–	–	–	–	38.7	25.1	32.2	44.6	29.0	–	35.0	36.0	36.1
	1-3 / month	–	–	–	–	–	–	5.4	2.7	7.3	5.0	8.3	–	3.3	5.7	5.7
	1-2 / week	–	–	–	–	–	–	4.3	2.3	8.0	4.6	8.3	–	6.1	5.7	5.6
	3-4 / week	–	–	–	–	–	–	9.3	8.6	9.7	8.1	12.9	–	7.2	9.5	9.5
	almost daily	–	–	–	–	–	–	42.2	61.3	42.7	37.8	41.4	–	48.3	43.1	43.1

Age adjusted**	Almost None	23.2	13.9	19.0	26.7	17.8	20.1	22.4	13.7	18.7	26.3	12.4	15.1	16.9	19.3	19.7
	1-3 / month	9.5	6.0	8.9	18.8	6.7	10.3	7.3	6.1	9.4	10.4	14.7	5.1	3.5	8.5	9.4
	1-2 / week	8.6	5.9	7.4	16.8	7.5	9.4	8.2	6.0	10.0	8.4	20.0	8.6	8.1	10.1	9.7
	3-4 / week	10.6	8.5	8.7	15.1	9.6	10.6	12.5	9.5	12.8	12.4	22.0	10.9	12.8	13.6	12.0
	almost daily	48.0	65.8	56.0	22.6	58.4	49.6	49.6	64.8	49.1	42.4	30.9	60.2	58.6	48.6	49.1

Ratio***	Almost None	1.2	0.7	1.0	1.4	0.9	1.0	1.1	0.7	0.9	1.3	0.6	0.8	0.9	1.0	1.0
	1-3 / month	1.0	0.6	0.9	2.0	0.7	1.1	0.8	0.6	1.0	1.1	1.6	0.5	0.4	0.9	1.0
	1-2 / week	0.9	0.6	0.8	1.7	0.8	1.0	0.8	0.6	1.0	0.9	2.1	0.9	0.8	1.0	1.0
	3-4 / week	0.9	0.7	0.7	1.3	0.8	0.9	1.0	0.8	1.1	1.0	1.8	0.9	1.1	1.1	1.0
	almost daily	1.0	1.3	1.1	0.5	1.2	1.0	1.0	1.3	1.0	0.9	0.6	1.2	1.2	1.0	1.0

* Study population consisted of only those aged 40 or 50 exactly in Katsushika and Suita1

** Age adjusted proportion by Japanese standard population (1985) using the data for age group 40-49 and 50-59

*** Ratio of the age-adjusted proportions setting that for 11 heath centers combined to be the denominator

For males, the proportion of those who drink almost daily was higher in Yokote, Kashiwazaki, Katsushika and Suita, and lower in Ishikawa and Miyako. The proportion of those who drink almost never was higher in Ishikawa and Arikawa and lower in Miyako, Kashiwazaki, and Yokote. In Ishikawa and Miyako, the proportion of those who drink occasionally was higher, while it was lower in Yokote and Kashiwazaki.

Table 4b shows the distribution of alcohol consumption in females by age and public health center. Overall, the age-adjusted proportion of those who drink almost daily was 5.9%, while that of those who drink almost never was 71.6%. In the older age group, the proportion of those who drink almost never was higher, while that of those who drink occasionally was lower and that of those who drink almost daily was lower than the younger ages.

**Table 4b.** Distribution for frequency of alcohol intake by age and health center, female.

		Cohort I (1990)						Cohort II (1993)								Total
	
Health Center		Ninohe	Yokote	Saku	Ishikawa	Katsushika*	Total	Kasama	Kashiwazaki	Tosayamada	Arikawa	Miyako	Suita1*	Suita2	Total	
Prefecture		Iwate	Akita	Nagano	Okinawa	Tokyo		Ibaraki	Niigata	Kochi	Nagasaki	Okinawa	Osaka	Osaka		
Age	Frequency															
40-49	Almost None	74.4	67.4	66.6	81.0	49.9	69.2	68.1	66.1	62.3	74.4	78.5	47.6	54.4	65.9	67.6
	1-3 / month	13.8	15.0	16.9	10.5	15.6	14.2	14.0	18.8	13.8	14.7	10.5	16.4	10.2	13.9	14.1
	1-2 / week	5.6	7.7	6.7	3.1	9.3	6.3	7.2	8.9	8.4	4.9	4.1	14.3	10.2	7.8	7.0
	3-4 / week	3.1	4.7	3.6	2.1	8.3	4.1	5.2	4.2	7.2	2.8	2.8	8.3	12.4	5.6	4.8
	almost daily	3.2	5.3	6.1	3.3	17.0	6.2	5.6	2.0	8.2	3.2	4.0	13.4	12.9	6.8	6.5

50-59	Almost None	84.1	77.2	73.4	90.9	55.2	76.5	78.4	84.4	76.3	88.4	91.1	53.3	64.6	76.9	76.7
	1-3 / month	9.4	10.3	14.8	5.0	13.6	10.7	9.3	8.4	10.1	5.6	4.0	14.4	6.6	8.5	9.7
	1-2 / week	2.9	4.3	4.9	1.8	9.1	4.5	4.4	2.8	5.5	2.3	1.3	10.3	9.2	5.0	4.7
	3-4 / week	1.5	3.3	2.7	1.2	8.5	3.3	3.8	2.9	4.2	1.8	1.5	9.1	8.6	4.4	3.8
	almost daily	2.0	4.9	4.2	1.1	13.6	5.0	4.0	1.5	4.0	1.9	2.1	12.9	10.9	5.3	5.1

60-69	Almost None	–	–	–	–	–	–	86.3	94.1	87.2	94.3	96.2	–	77.0	89.9	89.9
	1-3 / month	–	–	–	–	–	–	4.7	2.4	4.7	2.3	1.4	–	4.2	3.3	3.3
	1-2 / week	–	–	–	–	–	–	3.8	1.9	3.2	1.5	1.0	–	4.5	2.6	2.6
	3-4 / week	–	–	–	–	–	–	2.3	0.8	2.3	0.5	0.8	–	5.2	1.7	1.7
	almost daily	–	–	–	–	–	–	3.0	0.8	2.6	1.4	0.6	–	9.0	2.5	2.5

Age adjusted**	Almost None	78.7	71.7	69.6	85.4	52.2	72.4	72.6	74.2	68.5	80.6	84.0	50.1	58.9	70.7	71.6
	1-3 / month	11.9	12.9	16.0	8.1	14.7	12.7	11.9	14.2	12.2	10.7	7.6	15.5	8.6	11.5	12.2
	1-2 / week	4.4	6.2	5.9	2.5	9.2	5.5	6.0	6.2	7.1	3.8	2.9	12.5	9.8	6.6	6.0
	3-4 / week	2.4	4.1	3.2	1.7	8.4	3.7	4.6	3.6	5.9	2.4	2.2	8.7	10.7	5.1	4.4
	almost daily	2.7	5.1	5.3	2.3	15.5	5.7	4.9	1.8	6.4	2.6	3.2	13.2	12.0	6.1	5.9

Ratio***	Almost None	1.1	1.0	1.0	1.2	0.7	1.0	1.0	1.0	1.0	1.1	1.2	0.7	0.8	1.0	1.0
	1-3 / month	1.0	1.1	1.3	0.7	1.2	1.0	1.0	1.2	1.0	0.9	0.6	1.3	0.7	0.9	1.0
	1-2 / week	0.7	1.0	1.0	0.4	1.5	0.9	1.0	1.0	1.2	0.6	0.5	2.1	1.6	1.1	1.0
	3-4 / week	0.5	0.9	0.7	0.4	1.9	0.9	1.1	0.8	1.3	0.5	0.5	2.0	2.5	1.2	1.0
	almost daily	0.5	0.9	0.9	0.4	2.6	1.0	0.8	0.3	1.1	0.4	0.5	2.2	2.0	1.0	1.0

* Study population consisted of only those aged 40 or 50 exactly in Katsushika and Suita1

** Age adjusted proportion by Japanese standard population (1985) using the data for age group 40-49 and 50-59

*** Ratio of the age-adjusted proportions setting that for 11 heath centers combined to be the denominator

In females, the proportion of those who drink almost daily was higher in Katsushika and Suita, and lower in Kashiwazaki, Ishikawa, Arikawa, Nihohe and Miyako. In contrast, the proportion of those who almost never drink was higher in Ishikawa and Miyako and lower in Katsushika and Suita. The proportion of those who drink occasionally was higher in Katsushika, Suita, and Tosayamada.

Table 5a shows alcohol consumption by beverage type in males who drink any type of alcohol at least 1-2 times per week. People in the older age group tended to drink Japanese traditional alcohol beverages such as Japanese Sake, Shochu or Awamori more frequently, while those in the younger age group tended to drink westernized beverages such as beer, whiskey, brandy or vodka more frequently.

**Table 5a.** Proportion of people who drink at least once per week according to kind of alcohol beverage by age and heath center, male.

		Cohort I (1990)						Cohort II (1993)								Total
	
Health Center		Ninohe	Yokote	Saku	Ishikawa	Katsushika*	Total	Kasama	Kashiwazaki	Tosayamada	Arikawa	Miyako	Suita1*	Suita2	Total	
Prefecture		Iwate	Akita	Nagano	Okinawa	Tokyo		Ibaraki	Niigata	Kochi	Nagasaki	Okinawa	Osaka	Osaka		
Age	Type of alcohol															
40-49																
	Japanese sake	47.2	58.5	58.1	4.8	20.1	41.4	59.8	75.2	74.9	39.3	3.1	37.3	41.6	48.0	44.6
	Shochu, Awamori	27.6	9.4	19.2	43.1	18.8	22.6	20.5	4.1	9.1	35.0	84.0	15.0	12.8	28.0	25.3
	Beer	44.7	57.6	38.3	61.3	76.6	53.7	71.3	69.5	84.6	75.1	65.1	95.5	93.4	76.9	65.2
	Whiskey, Brandy, Vodka	20.2	13.7	15.9	24.8	23.3	18.7	19.0	20.8	26.6	33.3	9.1	43.9	39.7	24.8	21.7
	Wine	0.4	0.4	0.5	0.1	0.2	0.3	1.1	0.9	1.2	0.5	0.5	1.7	1.4	1.0	0.7
	Others	0.3	0.2	0.1	0.2	0.1	0.2	1.1	0.3	0.5	1.3	5.3	0.0	0.0	1.4	0.8

50-59																
	Japanese sake	51.5	67.7	63.7	4.6	25.8	48.0	66.9	83.6	80.8	42.4	2.6	45.1	49.0	51.0	49.4
	Shochu, Awamori	37.0	11.7	24.0	57.8	18.0	27.4	20.4	7.7	11.0	43.1	87.6	14.6	11.0	29.7	28.5
	Beer	24.8	37.8	23.3	46.2	69.4	37.7	56.6	48.3	70.7	53.9	50.0	90.8	84.2	65.1	50.6
	Whiskey, Brandy, Vodka	13.2	9.3	9.7	17.8	19.4	12.8	15.2	8.5	12.2	25.6	8.1	39.0	30.9	20.6	16.5
	Wine	0.2	0.3	0.2	0.2	0.1	0.2	0.8	0.5	1.1	1.3	0.6	0.9	1.3	0.9	0.5
	Others	0.0	0.2	0.2	0.4	0.4	0.2	1.1	1.9	1.8	1.3	3.2	0.1	0.6	1.3	0.7

60-69																
	Japanese sake	–	–	–	–	–	–	75.5	91.6	87.7	47.4	1.1	–	51.6	56.0	56.0
	Shochu, Awamori	–	–	–	–	–	–	15.5	6.9	14.0	51.8	85.9	–	12.0	34.8	34.8
	Beer	–	–	–	–	–	–	38.4	36.7	53.2	38.8	37.3	–	76.1	44.3	44.3
	Whiskey, Brandy, Vodka	–	–	–	–	–	–	9.3	3.2	6.0	9.5	4.8	–	22.8	9.0	9.0
	Wine	–	–	–	–	–	–	1.0	0.5	0.9	1.5	0.8	–	1.7	1.1	1.1
	Others	–	–	–	–	–	–	1.3	0.2	0.3	2.3	2.4	–	1.1	1.5	1.5

Age adjusted**																
	Japanese sake	49.1	62.6	60.6	4.7	22.6	44.3	62.9	78.9	77.5	40.7	2.9	40.7	44.9	49.3	46.7
	Shochu, Awamori	31.7	10.4	21.3	49.6	18.4	24.7	20.5	5.7	9.9	38.6	85.6	14.8	12.0	28.7	26.7
	Beer	35.9	48.9	31.7	54.7	73.4	46.7	64.8	60.2	78.5	65.8	58.5	93.4	89.3	71.7	58.8
	Whiskey, Brandy, Vodka	17.1	11.8	13.2	21.7	21.6	16.1	17.3	15.4	20.3	29.9	8.7	41.7	35.8	23.0	19.4
	Wine	0.3	0.4	0.4	0.1	0.2	0.3	1.0	0.7	1.2	0.9	0.5	1.3	1.4	1.0	0.6
	Others	0.2	0.2	0.1	0.3	0.2	0.2	1.1	1.0	1.1	1.3	4.4	0.0	0.3	1.4	0.8

Ratio***																
	Japanese sake	1.1	1.3	1.3	0.1	0.5	0.9	1.3	1.7	1.7	0.9	0.1	0.9	1.0	1.1	1.0
	Shochu, Awamori	1.2	0.4	0.8	1.9	0.7	0.9	0.8	0.2	0.4	1.4	3.2	0.6	0.4	1.1	1.0
	Beer	0.6	0.8	0.5	0.9	1.2	0.8	1.1	1.0	1.3	1.1	1.0	1.6	1.5	1.2	1.0
	Whiskey, Brandy, Vodka	0.9	0.6	0.7	1.1	1.1	0.8	0.9	0.8	1.0	1.5	0.4	2.2	1.8	1.2	1.0
	Wine	0.5	0.6	0.6	0.2	0.3	0.4	1.6	1.2	1.9	1.4	0.9	2.2	2.2	1.6	1.0
	Others	0.2	0.3	0.2	0.4	0.3	0.3	1.5	1.3	1.4	1.7	5.8	0.1	0.3	1.8	1.0

* Study population consisted of only those aged 40 or 50 exactly in Katsushika and Suita1

** Age adjusted proportion by Japanese standard population (1985) using the data for age group 40-49 and 50-59

*** Ratio of the age-adjusted proportions setting that for 11 heath centers combined to be the denominator

Alcohol consumption varied by public health center in males but not in females. In males, beer was the most popular type of alcohol beverage in Suita, Katsushika, Arikawa, Kasama, Ishikawa, while Japanese Sake was the most popular in Kashiwazaki, Yokote, and Saku. In Tosayamada, both beer and Japanese Sake were consumed frequently, while in Ninohe, both were infrequently consumed. In Miyako, Awamori was preferred to beer, while in Ishikawa, Awamori was consumed as frequently as beer. In both Miyako and Ishikawa, Japanese Sake was seldom consumed. In Arikawa, those in younger age groups preferred beer, while those in older age groups preferred Shochu. Whiskey, brandy and vodka were more frequently consumed in Suita.

Table 5b shows the proportion of those who drink at least once a week in females according to the types of alcohol beverages by age and health center. In females, beer was the most popular alcohol beverage in all health centers. The proportion of those who drink beer at least once a week was higher in Suita, Tosayamada, Miyako, Katsushika and Ishikawa and lower in Saku. Japanese Sake ranked second in Saku, Tosayamada, Kasama, Nihohe, Kashiwazaki and Yokote. In Ishikawa and Arikawa, whiskey/brandy/vodka were second, while in Miyako, Awamori was second.

**Table 5b.** Proportion of people who drink at least once per week according to kind of alcohol beverage by age and heath center, female.

		Cohort I (1990)						Cohort II (1993)								Total
	
Health Center		Ninohe	Yokote	Saku	Ishikawa	Katsushika*	Total	Kasama	Kashiwazaki	Tosayamada	Arikawa	Miyako	Suita1*	Suita2	Total	
Prefecture		Iwate	Akita	Nagano	Okinawa	Tokyo		Ibaraki	Niigata	Kochi	Nagasaki	Okinawa	Osaka	Osaka		
Age	Type of alcohol															
40-49																
	Japanese sake	21.4	18.1	29.2	1.3	7.1	15.7	25.2	17.6	24.9	10.8	2.9	13.8	16.7	18.2	17.1
	Shochu, Awamori	9.2	4.1	8.6	11.0	13.4	9.3	13.3	0.0	2.3	8.1	27.4	4.9	5.6	8.9	9.1
	Beer	69.1	68.0	54.9	74.4	78.4	69.2	71.3	70.6	83.0	74.6	78.9	89.0	88.8	80.3	75.2
	Whiskey, Brandy, Vodka	13.7	16.8	18.6	42.3	14.1	19.0	13.4	14.7	14.7	29.7	12.0	16.7	15.1	15.9	17.3
	Wine	1.9	3.4	2.7	0.4	1.4	2.1	5.4	2.9	3.4	5.9	2.9	9.1	5.2	5.8	4.1
	Others	1.9	3.4	1.7	0.9	0.2	1.6	3.7	13.2	4.0	6.5	7.4	3.5	0.4	4.1	3.0

50-59																
	Japanese sake	36.2	27.7	45.2	5.1	11.8	23.8	31.6	41.0	38.0	27.3	6.0	18.4	17.1	23.6	23.7
	Shochu, Awamori	15.4	4.1	8.0	17.3	14.3	10.9	13.4	2.6	4.2	10.9	41.7	4.0	3.3	8.4	9.6
	Beer	47.7	62.3	42.4	73.5	69.8	61.0	67.8	61.5	75.9	60.9	78.6	88.8	87.5	79.5	70.2
	Whiskey, Brandy, Vodka	8.7	9.0	8.3	27.6	19.2	14.2	8.5	7.7	6.0	18.0	9.5	14.9	17.1	12.7	13.5
	Wine	2.0	2.8	6.7	3.1	1.6	3.0	4.3	5.1	6.6	7.8	7.1	9.0	6.3	7.1	5.0
	Others	3.4	2.6	1.9	2.0	0.1	1.5	4.0	7.7	4.2	7.0	2.4	2.5	0.8	3.2	2.3

60-69																
	Japanese sake	–	–	–	–	–	–	42.5	31.8	51.5	27.5	0.0	–	21.5	33.9	33.9
	Shochu, Awamori	–	–	–	–	–	–	5.5	0.0	3.9	21.3	22.7	–	1.3	7.1	7.1
	Beer	–	–	–	–	–	–	50.8	50.8	55.3	52.5	70.5	–	84.2	61.7	61.7
	Whiskey, Brandy, Vodka	–	–	–	–	–	–	4.7	4.7	3.9	3.8	4.5	–	4.5	4.5	4.5
	Wine	–	–	–	–	–	–	6.7	6.7	5.8	6.3	4.5	–	5.7	5.7	5.7
	Others	–	–	–	–	–	–	6.3	6.3	9.7	5.0	9.1	–	5.9	5.9	5.9

Age adjusted**																
	Japanese sake	27.9	22.3	36.2	3.0	9.2	19.3	28.0	27.9	30.7	18.1	4.3	15.8	16.9	20.6	20.0
	Shochu, Awamori	11.9	4.1	8.3	13.8	13.8	10.0	13.3	1.1	3.1	9.3	33.7	4.5	4.6	8.7	9.3
	Beer	59.7	65.5	49.4	74.0	74.6	65.6	69.8	66.6	79.9	68.6	78.8	88.9	88.2	79.9	73.0
	Whiskey, Brandy, Vodka	11.5	13.4	14.1	35.8	16.3	16.9	11.2	11.6	10.9	24.5	10.9	15.9	16.0	14.5	15.6
	Wine	1.9	3.1	4.5	1.6	1.5	2.5	4.9	3.9	4.8	6.7	4.7	9.1	5.7	6.4	4.5
	Others	2.6	3.0	1.8	1.4	0.2	1.6	3.8	10.8	4.1	6.7	5.2	3.1	0.6	3.7	2.7

Ratio***																
	Japanese sake	1.4	1.1	1.8	0.1	0.5	1.0	1.4	1.4	1.5	0.9	0.2	0.8	0.8	1.0	1.0
	Shochu, Awamori	1.3	0.4	0.9	1.5	1.5	1.1	1.4	0.1	0.3	1.0	3.6	0.5	0.5	0.9	1.0
	Beer	0.8	0.9	0.7	1.0	1.0	0.9	1.0	0.9	1.1	0.9	1.1	1.2	1.2	1.1	1.0
	Whiskey, Brandy, Vodka	0.7	0.9	0.9	2.3	1.0	1.1	0.7	0.7	0.7	1.6	0.7	1.0	1.0	0.9	1.0
	Wine	0.4	0.7	1.0	0.4	0.3	0.6	1.1	0.9	1.1	1.5	1.1	2.0	1.3	1.4	1.0
	Others	1.0	1.1	0.7	0.5	0.1	0.6	1.4	4.0	1.5	2.5	1.9	1.1	0.2	1.4	1.0

* Study population consisted of only those aged 40 or 50 exactly in Katsushika and Suita1

** Age adjusted proportion by Japanese standard population (1985) using the data for age group 40-49 and 50-59

*** Ratio of the age-adjusted proportions setting that for 11 heath centers combined to be the denominator

Table 6a shows the estimated amount of ethanol intake among those who drink at least once a week in the areas of Cohort 1, by sex, age and health center. The age-adjusted average amount of ethanol intake was 301g/week in males and 127g/week in females. The average amount of ethanol intake did not differ by age in both sexes. When compared by public health center, the average amount of ethanol intake was higher in Yokote and lower in Ishikawa for males, but higher in Ishikawa and lower in Saku and Yokote for females.

**Table 6a.** Average amount of alcohol intake (ethanol g/week) by sex, age and health center in Cohort I .

		Cohort I (1990)					Total

Health Center		Ninohe	Yokote	Saku	Ishikawa	Katsushika*	
Prefecture		Iwate	Akita	Nagano	Okinawa	Tokyo	
Male	40-49	320.2	334.4	286.1	254.1	300.0	300.7
	50-59	318.2	317.1	302.4	268.1	285.1	301.2
	60-69	–	–	–	–	–	–

	Age adjusted**	319.3	326.8	293.3	260.3	293.4	301.0
	Ratio***	1.06	1.09	0.97	0.86	0.98	1.00

Female	40-49	113.7	111.0	105.4	245.6	120.5	128.8
	50-59	128.2	113.4	85.0	184.3	140.0	124.7
	60-69	–	–	–	–	–	–

	Age adjusted**	120.1	112.1	96.4	218.6	129.1	127.0
	Ratio***	0.95	0.88	0.76	1.72	1.02	1.00

* Study population consisted of only those aged 40 or 50 exactly in Katsushika

** Age adjusted proportion by Japanese standard population (1985) using the data for age group 40-49 and 50-59

*** Ratio of the age-adjusted proportions setting that for 5 heath centers combined to be the denominator

Table 6b shows the estimated amount of ethanol intake, both from regular drinking and drinking at social events combined, among those who drink at least once per week in Cohort 2, by sex, age and public health center. The age-adjusted average amount of ethanol intake was 387.3g/week in males and 142.1g/week in females. In both sexes, the average amount of ethanol intake was lower in the age group of 60-69. The average amount of ethanol intake was higher in Miyako and lower in Kashiwazaki for both males and females and low in Suita among males.

**Table 6b.** Average amount of alcohol intake (ethanol g/week) by sex, age and health center in Cohort II.

		Cohort II (1993)							Total

Health Center		Kasama	Kashiwazaki	Tosayamada	Arikawa	Miyako	Suita1*	Suita2	
Prefecture		Ibaraki	Niigata	Kochi	Nagasaki	Okinawa	Osaka	Osaka	
Male	40-49	351.8	340.0	365.3	410.4	642.5	309.7	312.7	392.6
	50-59	355.5	315.9	359.1	393.3	539.7	359.4	288.2	380.6
	60-69	297.8	276.4	297.2	350.5	405.4	–	240.1	320.1

	Age adjusted**	353.4	329.4	362.6	402.9	597.2	331.6	301.9	387.3
	Ratio***	0.91	0.85	0.94	1.04	1.54	0.86	0.78	1.00

Female	40-49	160.1	79.2	121.9	132.9	347.4	107.5	112.0	145.0
	50-59	136.4	95.1	124.3	125.0	387.1	126.0	110.3	138.3
	60-69	110.8	136.3	115.4	125.5	136.8	–	84.0	109.1

	Age adjusted**	149.7	86.2	123.0	129.4	364.9	115.6	111.3	142.1
	Ratio***	1.05	0.61	0.87	0.91	2.57	0.81	0.78	1.00

* Study population consisted of only those who aged 40 or 50 exactly in Suita1

** Age adjusted proportion by Japanese standard population (1985) using the data for age group 40-49 and 50-59

*** Ratio of the age-adjusted proportions setting that for 6 heath centers combined to be the denominator

Table 6c shows the proportion of ethanol intake estimated from drinking at social events in Cohort 2, by sex, age and health center. Social drinking accounted for 26.1% and 17.4% of ethanol intake in males and females, respectively. In males, the age-adjusted proportion was higher in Miyako and lower in Kashiwazaki and Kasama, while in females, it was higher in Miyako and Tosayamada and lower in Kashiwazaki.

**Table 6c.** Proportion of alcohol intake (ethanol g/week) derived from drinking at social events by sex, age and health center in Cohort II.

		Cohort II (1993)							Total

Health Center		Kasama	Kashiwazaki	Tosayamada	Arikawa	Miyako	Suita1*	Suita2	
Prefecture		Ibaraki	Niigata	Kochi	Nagasaki	Okinawa	Osaka	Osaka	
Male	40-49	23.4%	21.9%	28.9%	25.6%	36.4%	29.0%	28.8%	28.0%
	50-59	18.8%	14.4%	26.6%	18.8%	29.9%	27.5%	23.6%	23.5%
	60-69	11.9%	14.2%	17.7%	11.6%	17.9%	–	15.8%	14.6%

	Age adjusted**	21.4%	18.6%	27.9%	22.6%	33.6%	28.3%	26.5%	26.1%
	Ratio***	0.82	0.71	1.07	0.87	1.29	1.09	1.02	1.00

Female	40-49	17.8%	8.2%	20.3%	13.3%	24.7%	18.6%	13.1%	18.6%
	50-59	13.4%	12.4%	18.3%	15.6%	16.7%	17.3%	13.1%	15.8%
	60-69	7.9%	20.6%	9.6%	11.0%	19.3%	–	6.3%	9.7%

	Age adjusted**	15.9%	10.1%	19.4%	14.3%	21.2%	18.0%	13.1%	17.4%
	Ratio***	0.91	0.58	1.12	0.82	1.22	1.04	0.75	1.00

* Study population consisted of only those aged 40 or 50 exactly in Suita1

** Age adjusted proportion by Japanese standard population (1985) using the data for age group 40-49 and 50-59

*** Ratio of the age-adjusted proportions setting that for 5 heath centers combined to be the denominator

## DISCUSSION

### Comparison to the nation-wide surveys

#### Smoking:

There are two main nation-wide surveys which monitor smoking behavior in the Japanese population^1^^, ^^2^^)^. In Table 7 the smoking data from the National Nutrition Survey and Japan Tobacco Survey are reported for comparison purposes. The proportion of current smokers reported by the Japan Tobacco Survey appears systematically higher than that reported by the National Nutrition Survey. This may be partly because the Japan Tobacco Survey includes occasional smokers as current smokers, while the National Nutrition Survey includes only regular smokers as current smokers. Our definition follows the approach of the National Nutrition Survey. The proportion of former smokers has not been reported by the Japan Tobacco Survey, though repeated surveys reveal a decreasing trend in current smokers since the late 1960s. In contrast, the proportion of female smokers has been rather constant. Because of the fluctuations in the annual smoking estimates for the Japan Tobacco Survey for 1990 to 1993, the average proportion in 1990-93 was calculated and the data from the National Nutrition Survey was used as a reference.

**Table 7.** Proportion of current and former smoker from other nation-wide surveys.

		National Nutrition Survey					Japan Tobacco Survey					Cohort I 1990	Cohort II 1993	Cohort I and II combined
	
		1990	1991	1992	1993	1990-93	1990	1991	1992	1993	1990-93			
Male														
Proportion of current smoker														
	40-49	56.6	55.6	55.6	47.3	53.8	62.7	64.3	65.1	65.6	64.4	57.1	59.3	58.2
	50-59	50.1	47.0	47.2	47.4	47.9	57.0	57.3	53.4	53.9	55.4	49.7	51.2	50.4
	60-69	51.8	52.4	48.1	41.8	48.5	49.4	51.3	49.2	46.7	49.2	–	43.9	43.9

	Age adjusted*	53.7	51.8	51.9	47.3	51.2	60.2	61.2	59.9	60.4	60.5	53.8	55.7	54.8
	Ratio**	1.05	1.01	1.01	0.92	1.00	1.18	1.20	1.17	1.18	1.18	1.05	1.09	1.07

Proportion of former smoker														
	40-49	16.7	13.9	15.3	15.7	15.4	–	–	–	–	–	20.6	19.7	19.7
	50-59	20.1	19.0	20.8	18.4	19.6	–	–	–	–	–	26.1	22.5	24.4
	60-69	30.1	27.5	28.8	29.0	28.9	–	–	–	–	–	–	34.7	34.7

	Age adjusted*	18.2	16.1	17.7	16.9	17.2	–	–	–	–	–	23.0	20.9	21.8
	Ratio**	1.06	0.94	1.03	0.98	1.00	–	–	–	–	–	1.34	1.21	1.26

Female														
Proportion of current smoker														
	40-49	11.3	11.8	11.1	11.7	11.5	14.0	13.7	12.6	13.0	13.3	9.2	9.8	9.5
	50-59	8.0	8.0	6.6	7.9	7.6	12.2	11.6	11.8	10.8	11.6	6.6	7.0	6.8
	60-69	8.5	7.0	6.9	6.1	7.1	9.4	9.7	8.2	8.2	8.9	–	4.6	4.6

	Age adjusted*	9.8	10.1	9.1	10.0	9.8	13.2	12.8	12.2	12.0	12.6	8.1	8.6	8.3
	Ratio**	1.01	1.04	0.93	1.03	1.00	1.35	1.31	1.25	1.23	1.28	0.82	0.88	0.85

Proportion of former smoker														
	40-49	1.4	1.6	1.6	1.6	1.6	–	–	–	–	–	3.0	1.7	2.4
	50-59	1.4	1.6	1.8	1.6	1.6	–	–	–	–	–	2.2	1.3	1.8
	60-69	3.0	2.4	2.3	1.8	2.4	–	–	–	–	–	–	1.6	1.6

	Age adjusted*	1.4	1.6	1.7	1.6	1.6	–	–	–	–	–	2.6	1.5	2.1
	Ratio**	0.89	1.02	1.07	1.02	1.00	–	–	–	–	–	1.68	0.97	1.36

* Age adjusted proportion by Japanese standard population (1985) using the data for age group 40-49 and 50-59

** Ratio of the age-adjusted proportions setting that for average proporion of National Nutrition Survey from 1990 to 1993 to be the denominator

Compared to the National Nutrition Survey, the proportion of current smokers and former smokers in this study population was slightly higher in males except for the age group of 60-69 years. In females, the estimated proportion of current smokers from our study was slightly lower for all age groups, compared to the National Nutrition Survey, while the estimate of former smokers was slightly higher except for the age group of 60-69 years. According to the Japan Tobacco Survey, the proportion of current smokers in males did not vary remarkably by area. In females, however, the proportion of current smokers in Hokkaido was higher than 20%, which was 2 times higher than the other geographic areas. Since this study did not cover Hokkaido, this would be one explanation for the discrepancies in estimates.

#### Drinking:

The National Nutrition Survey also reports on a frequency of alcohol consumption of 3 days or more per week and 1 “go” (180ml of Japanese Sake or equivalent amount of alcohol) or more per day^2^^)^. In Table 8, the proportion of habitual drinkers observed in the National Nutrition Survey in 1990-93 is shown alongside the estimates from our study. During 1990-93, the proportion declined slightly in males, but remained fairly constant in females. Compared to the average proportion of drinkers in 1990-93 observed in the National Nutrition Survey, Cohort I showed no difference in males and a lower proportion of drinkers in females, while Cohort 2 tended to be higher for both sexes. This is mainly because drinking at social events was specifically queried in Cohort II, while in the National Nutrition Survey and Cohort I, this was not specifically considered.

**Table 8.** Proportion of habitual drinker* from other nation-wide surveys.

		National Nutrition Survey					Cohort I 1990	Cohort II 1993	Cohort I and II combined

		1990	1991	1992	1993	1990-1993			
Male	40-49	60.7	58.4	57.1	52.7	57.2	57.3	61.4	61.1
	50-59	59.1	55.2	60.1	53.8	57.1	57.0	61.5	61.2
	60-69	55.1	50.1	54.7	49.1	52.3	–	51.7	52.6

	Age adjusted*	60.0	57.0	58.4	53.2	57.1	57.2	61.4	61.1
	Ratio**	1.05	1.00	1.02	0.93	1.00	1.00	1.08	1.07

Female	40-49	8.9	10.1	10.5	9.9	9.9	7.1	12.0	11.3
	50-59	6.3	6.2	7.1	6.8	6.6	5.4	9.4	8.9
	60-69	4.8	5.0	4.0	3.2	4.3	–	4.0	4.2

	Age adjusted*	7.8	8.4	9.0	8.5	8.4	6.4	10.9	10.2
	Ratio**	0.92	1.00	1.07	1.01	1.00	0.75	1.29	1.22

* Habitual drinker was defined as those who drink alcohol 3 days or more per week and 1 go or more per day

** Age adjusted proportion by Japanese standard population (1985) using the data for age group 40-49 and 50-59

*** Ratio of the age-adjusted proportions setting that for average proporion of National Nutrition Survey from 1990 to 1993 to be the denominator

#### Methodological considerations:

This analysis has some methodological limitations that need to be discussed. First, the proportion of former smokers appears systematically lower in Cohort II. This may be bias due to the different questionnaire used in Cohort II. In Cohort II, if former smokers did not provide detailed information on their previous smoking habits, such as average number of cigarette smoked per day or age at initiation of smoking, then they were considered non smokers, while in Cohort I, former smokers were queried directly as to their current and former smoking status. Therefore, the proportion of former smokers in Cohort II may be underestimated and caution should be applied when interpreting the findings.

Second, the amount of ethanol intake may be underreported in Cohort I, because social drinking was not specifically queried as in Cohort II. This systematic underreporting would be of particular concern in geographic areas where social drinking constitutes a large proportion of ethanol intake as may exist in Ishikawa. For most previous studies, however, drinking at social events has seldom been asked. There is a possibility that people do not clearly differentiate between regular drinking or drinking at social events and might report the frequency of both types of drinking combined. This may result in the amount of intake being overestimated in Cohort II. Because the responses in regular drinking might be affected by the different structures of the questionnaires, comparison of alcohol intake in Cohort I and II was limited to the frequency of alcohol intake. Estimated amount of alcohol intake was compared separately. This problem should be further investigated by other dietary survey approaches, such as dietary records for selected subjects.

Third, ethanol intake may be overestimated in Miyako and Ishikawa, because of possible confusion whether alcohol intake should be reported by the volume of spirits with or without water. In calculating ethanol intake, such as Awamori or Shochu, we considered intake to be without water, but in the questionnaire there was no clear indication on this point. Because many people like to drink strong spirits diluted with water, people may report the volume of spirits with water instead of without in the questionnaire. This would lead to overestimation of the intake of ethanol especially in Miyako and Ishikawa, where a considerable proportion of alcohol intake is derived from spirits. Again, this should be further investigated by other dietary survey approaches.

### Internal Comparison

#### Smoking:

The proportion of current male smokers did not vary remarkably by health center except for lower proportions observed in Ishikawa and Miyako, both part of the Okinawa Prefecture. In Ishikawa, since the proportion of former smokers was relatively high, the proportion of ever smokers was accordingly high at 71.0%. In Miyako, however, the proportion of former smokers was low, but the proportion of ever smokers was 55.3%, which was low relative to other public health centers. In contrast to the lower smoking rates, lung cancer mortality in the Okinawa Prefecture is the highest in Japan in both males and females. These findings seem to be contradictory, since cigarette smoking is the most influential risk factor for lung cancer. In terms of the production and sales of cigarettes, however, Okinawa Prefecture has a special history, different from other prefectures in Japan. During 1945-72, while Okinawa was governed by the US, Okinawa had an original production and sales system of cigarettes. The original brands of cigarettes, which contain relatively high tar and nicotine, are still on sale only in Okinawa Prefecture. Such ecological comparisons may be misleading because we are measuring current smoking habits in relation to lung cancer risk which has a long latency.

In females, the proportion of current smokers appeared to be highly dependent on the extent of urbanization. There was a five-fold difference on the proportion of current smokers between Katsushika (20%) and Miyako (4%). These findings are consistent with the geographical variation of lung cancer mortality, which shows higher rates in urban areas. Again, the exception is Okinawa Prefecture, where lung cancer mortality in females is also the highest in Japan while the smoking frequency is low.

#### Drinking:

In males, the proportion of those who drink daily was higher in Yokote and Kashiwazaki, both located in areas where production of Japanese Sake is prospering. In urban areas, such as Katsushika and Suita, the proportion of daily drinkers is higher as well. In Ishikawa and Miyako, people tend to drink less frequently, such as 1-2 times per week. This may be because in Yokote and Kashiwazaki, people prefer to drink at home with dinner, while in Ishikawa and Miyako, people prefer to drink outside of the home. The average number of days of social drinking among those who drink at least once per week in males was 6.2 in Miyako, while it was 2.8 in Kashiwazaki. Since the amount of alcohol intake is usually higher when drinking together, the total amount of alcohol intake was higher in Miyako. In terms of total alcohol intake, for Miyako 33.5% was from drinking at social events, while for Kashiwazaki, the figure was only 18.6%. However, the estimated amount of alcohol intake may be overestimated in Miyako and Ishikawa.

In females, the proportion of daily drinkers was highly dependent on the extent of urbanization, similar to smoking habits. In Katsushika and Suita, 12% to 15% of women were daily drinkers, while in Kashiwazaki, Ishikawa, Arikawa and Nihohe, it was less than 3%. In Tosayamada, the proportion of daily drinkers combined with the occasional drinkers was relatively higher than the other remaining areas. This may be due to the fact that the local social norm in Tosayamada has not been so strict against women drinking.

There was a great variation on the types of alcohol beverages frequently consumed in males, while there was little variation in females. In Yokote, Saku, and Kashiwazaki, which are located in a relatively cold climate and also famous for the production of Japanese Sake, people like to drink Japanese Sake, especially among the older age group. In Katsushika and Suita, both belonging to urban areas, or Ishikawa, Tosayamada, Arikawa and Miyako, which are located in warm or moderate climates, beer was the most popular alcoholic beverage. In Miyako, where the production of Awamori, a traditional distilled liquor made from rice, is prosperous, people like to drink Awamori most, while in Ishikawa, people like to drink both Awamori and beer. Shochu, which is also a type of a traditional distilled liquor, but mainly made from wheat, sweet potatoes or buckwheat, is frequently consumed in Arikawa and Ninohe, where there is no production of an original local alcohol beverage. Whiskey, brandy or vodka was popular in urban areas like Suita. In all geographic areas, women tend to consume beer most frequently.

In summary, smoking and drinking habits in this study population were comparable to patterns observed in other nationwide surveys in Japan. At the same time, however, considerable variations in smoking and drinking habits were observed according to health center. Some differences were consistent with previous knowledge, but some of them were not. In order to clarify exposure and disease relationships in these health centers, future analyses will consider not only smoking and drinking habits but also other various life-style factors.
